# California’s Comprehensive Perinatal Services Program and birth outcomes

**DOI:** 10.3389/fpubh.2023.1321313

**Published:** 2023-12-21

**Authors:** Snehal S. Lopes, Ahan Shi, Liwei Chen, Jian Li, Laurie L. Meschke

**Affiliations:** ^1^Department of Public Health Sciences, Clemson University, Clemson, SC, United States; ^2^D.W. Daniel High School, Central, SC, United States; ^3^Department of Epidemiology, Fielding School of Public Health, University of California, Los Angeles, Los Angeles, CA, United States; ^4^Fielding School of Public Health, Environmental Health Sciences Department and the School of Nursing, University of California, Los Angeles, Los Angeles, CA, United States; ^5^Department of Public Health, University of Tennessee, Knoxville, TN, United States

**Keywords:** Comprehensive Perinatal Service Program (CPSP), Medicaid, spontaneous preterm birth (SPTB), preterm birth (PTB), low birthweight (LBW)

## Abstract

**Introduction:**

California’s Medicaid (Medi-Cal) sponsors Comprehensive Perinatal Services Program (CPSP), a program with enhanced perinatal care for women (more education, nutritional supplements, and psychosocial counseling/support). Past evaluations of CPSP’s effectiveness in birth outcomes were limited to pilot programs and yielded mixed results.

**Methods:**

We used 2012–2016 California’s statewide data about singleton live births with any receipt of prenatal care (*N* = 2,385,811) to examine whether Medi-Cal with CPSP enrollment was associated with lower odds of preterm birth (PTB), spontaneous PTB, and low birthweight (LBW) than non-CPSP births. With three binary variables of PTB, spontaneous PTB, and LBW as the response variables, three multilevel logistic models were used to compare the outcomes of participants enrolled in Medi-Cal with CPSP against those with private insurance, adjusting for maternal factors and county-level covariates.

**Results:**

Logistic models showed that participants enrolled to Medi-Cal with CPSP [*n* (%) = 89,009 (3.7)] had lower odds of PTB, spontaneous PTB and LBW, respectively, as compared with those with private insurance [*n* (%) = 1,133,140 (47.2)]. Within the Medi-Cal sub-population, the CPSP enrollment was associated with lower odds of PTB, SPTB and LBW than Medicaid beneficiaries without CPSP [*n* (%) = 967,094 (40.3)].

**Discussion:**

With statewide data, these findings revealed a robust link between CPSP enrollment and better birth outcomes. Expanding access to comprehensive prenatal services could be an important strategy to improve birth outcomes.

## Introduction

Access to perinatal services is critical for maternal health and birth outcomes (1, 2), and a society’s investment in evidence-based perinatal service has been shown to have favorable long-term cost-effectiveness ratio (3). The persistent racial/ethnic disparities in birth outcomes (4) have made it particularly an urgent task for public health stakeholders to identify evidence-based perinatal care models, as the negative impact of adverse birth outcomes on one’s cognitive (5), neurodevelopmental (6), and physical health outcomes (7, 8) throughout the life course indicates that the racial disparity in birth outcomes has broader significance for racial justice in a society. So far, care models for reducing preterm births (PTB) include group antenatal care (9), enhanced/targeted care for HIV-positive women (10, 11), and augmented care for women with overall elevated risk (more educational sessions, additional appointments, extended time with clinicians, etc.) (12, 13). However, relatively few prenatal service programs have been evaluated with statewide data with regard to birth outcomes such as PTB and low birthweight (LBW), both of which incur substantial burden of illness and disproportionately affect minority populations (14).

As one of the earlier state-level programs to improve maternal health and birth outcomes in the United States, the California Medicaid program (Medi-Cal) offers the voluntary enrollment Comprehensive Perinatal Services Program (CPSP) to provide enhanced services such as psychosocial counseling, nutritional supplements, and health education (15, 16). These services are provided by a team of professional which includes physicians, certified nurse midwives, certified nutritionists, social workers with a master’s level training, and certified nurse educators (1). The CPSP benefit has been offered through Medi-Cal since 1987, and is an offshoot of the Obstetrical Access Project (1979–1982) that operated in 13 counties in California (17). All pregnant women eligible for Medi-Cal are eligible to receive the CPSP service (17). The income threshold for Medi-Cal eligibility is higher for pregnant women (up to 213% of federal poverty level) (17). Pregnant women without satisfactory immigration status verification may also qualify for Medi-Cal (17). All Medi-Cal managed health plans are required to ensure that their pregnant enrollees have access to the CPSP services (17). Data on the implementation and utilization of the CPSP is limited, and access to the program may not be optimal (17). For the psychosocial support part, CPSP mandates assessments of the woman’s psychosocial status at baseline once prenatal care has been initiated and during each trimester, combined with a care plan to prevent or address psychosocial challenges with treatment referrals when appropriate (16). So far, evaluations of CPSP’s impact on birth outcomes yielded inconclusive results (1, 18). The 1995 study demonstrating CPSP’s better birth outcomes than the alternate care (18) was based upon a pilot program, not statewide sample. Hence, it is important to provide an updated assessment of CPSP’s link with birth outcomes using statewide data.

In this study, we used California’s Birth Statistical Master Files (BSMF) (19) 2012–2016, a source registering all live births (20), to investigate whether one’s CPSP enrollment was associated with the odds of PTB and LBW. As BSMF documents whether a preterm birth case is a case of spontaneous preterm birth (SPTB), the kind of preterm that is both most prevalent and the least understood (21), we choose to examine the link between CPSP enrollment and SPTB separately, in addition to our study of the link between CPSP enrollment and PTB in general.

## Methods

### Data, study design and population

We conducted a pooled cross-sectional study using California’s BSMF data (19) from 2012 to 2016, with ethical approvals from Clemson University, the Committee for the Protection of Human Subjects (CPHS), and the Vital Statistics Advisory Committee (VSAC). We included women residents of California State with singleton births between 2012 and 2016 who received any prenatal care (*N* = 2,385,811). We used a multilevel model in our analysis with the county as the cluster level, given the documented significant association between individual-level birth outcomes and county-level factors in the United States (22–24) [including California (24)].

### Exposure

The exposure variable was the payment source for prenatal care (private insurance, Medi-Cal with CPSP, Medi-Cal without CPSP, no prenatal care, other government programs, private insurance, self-pay, other unknown/unreported source). The BMSF data for prenatal care payment source records Medi-Cal with CPSP and Medi-Cal without CPSP enrollment as separate categories.

### Outcomes

We had three outcome variables: PTB (<37 weeks of gestation), spontaneous PTB [defined as a birth prior to 37 weeks of gestation with spontaneous labor or membrane rupture (14), use of tocolysis (25), or cervical cerclage (26)], and LBW (birthweight less than 2,500 grams) (27). The gestational age and birthweight were recorded by hospital staff upon birth.

### Covariates

Individual-level maternal factors we used to adjust for confounding effects included race/ethnicity, age, educational attainment, pre-pregnancy and gestational diabetes, pre-pregnancy and gestational hypertension, cigarette smoking (yes/no) during pregnancy, pre-pregnancy BMI, parity (categorized as 1, 5–9, 10+ births), month of birth (as a proxy for the seasonality factor) (28), and year of birth (as a proxy for the secular trend of PTB and LBW) (29), as these individual-level factors have been documented as related with birth outcomes.

As for county-level environmental factors, we used county-level covariates including average unemployment rate for the 12 months prior to birth (30) [obtained from Bureau of Labor Statistics (31)], 5-year average proportion of foreign born population prior to year of birth (32) [obtained from County Health Ranking (33)], and 5-year average proportion of non-English speakers prior to year of birth (32) [obtained from County Health Ranking (33)], as the local unemployment rate and immigrant density are known environmental factors that influence birth outcomes (34, 35).

### Statistical analyses

Descriptive statistics were calculated for the characteristics of the study population and the characteristics were compared between three prenatal care payment sources (private insurance, Medi-Cal with CPSP, Medi-Cal without CPSP). Bivariate analyses were conducted to explore the distribution of the outcomes across different sources for prenatal care. We used three multilevel logistic regression analyses with random intercept for the cluster variable (county) to investigate the association between CPSP coverage and the outcomes: overall PTB (non-PTB as reference), spontaneous PTB (not spontaneous PTB as reference), and LBW (≥2,500 grams as reference), while the county of maternal residence was used as the Level 2 cluster (30). For the exposure variable in these three multilevel models, the cases with private insurance were used as the reference group, as private insurance beneficiaries had been shown as having relatively low prevalence of adverse birth outcomes (36).

To understand whether the association between CPSP enrollment and birth outcomes varied across racial/ethnic groups [given the racial disparity in birth outcomes (14)], we conducted multilevel analyses of the Medi-Cal-covered cases, including both those receiving CPSP and those not receiving CPSP. The exposure variable for this subsample analysis was a binary variable indicating whether the prenatal care was supported by CPSP. Additional analyses were conducted for the three multilevel models using the Medi-Cal subsample: (1) including the interaction terms between race/ethnicity and CPSP where a significant interaction term would indicate that the association between CPSP and birth outcome was different between a racial/ethnic group and the reference group of non-Hispanic White persons; (2) using inverse probability of treatment weighting [IPTW (37), treatment here is CPSP] using propensity scores to adjust for self-selection bias. The GLIMMIX procedure in SAS 9.4 was used to conduct the multilevel analyses.

## Results

A total of 2,385,811 live singleton births in California from 2012 to 2016 were included (Table 1), with private insurance covering 47.5%, Medi-Cal cases without CPSP constituting 40.5%, and Medi-Cal-CPSP constituting 3.7% of the total sample. PTB made up 7.1% of the sample, with SPTB and LBW accounting for 4.5 and 5.2%, respectively. Persons from the Hispanic ethnicity made up 48.0% of the statewide sample, while other major racial/ethnic groups included persons from non-Hispanic White race (27.2%), East Asian race (6.0%), Southeast Asian race (5.1%), Black race (5.0%), South Asian race (2.4%) and mixed race (2.7%).

**Table 1:** Descriptive statistics of 2,385,811 singleton births and the socio-economic environment factors in California 2012–2016.

	Total sample		Private insurance		Medi-Cal with CPSP		Medi-Cal without CPSP		*p* ^e^
	Mean	SD	Mean	SD	Mean	SD	Mean	SD	
County level variables									
Unemployment ^a^ (%)	8.7	3.2	8.3	3.0	9.8	3.6	9.2	3.2	–
Foreign born ^b^ (%)	27.1	7.4	27.4	7.4	21.7	7.6	27.0	7.3	–
Non-English speakers ^c^ (%)	44.1	10.9	43.6	10.7	37.4	12.6	45.1	10.6	–
Individual level variables									
Maternal age (years)	29.2	6.1	31.0	5.4	26.9	6.0	27.2	6.1	<0.001
Pre-pregnancy BMI (kg/m^2^)	26.2	6.2	25.5	5.9	27.4	6.6	27.1	6.4	<0.001
Birth weight (grams)	3327.4	538.0	3349.1	532.9	3347.1	529.6	3299.9	546.8	<0.001
Gestational age (days)	275.1	18.3	275.8	18.1	275.9	17.7	274.3	18.0	<0.001
	N	% (column percent)	N	% (column percent)	N	% (column percent)	N	% (column percent)	
Race/ethnicity									<0.001
Non-Hispanic White	649,512	27.2	448,051	39.5	19,025	21.4	127,733	13.2	
Black	118,365	5.0	40,115	3.5	4,586	5.2	61,836	6.4	
American Indian	8,029	0.3	2,716	0.2	835	0.9	3,541	0.4	
Other/unknown/refused	56,184	2.4	35,497	3.1	914	1.0	15,306	1.6	
Pacific islander^d^	9,079	0.4	3,839	0.3	416	0.5	4,008	0.4	
Mixed race	64,068	2.7	37,981	3.4	2,593	2.9	18,891	2.0	
Asian other	13,778	0.6	9,042	0.8	508	0.6	3,375	0.4	
Asian-South	57,769	2.4	46,744	4.1	739	0.8	7,586	0.8	
Asian-South-East	120,486	5.1	76,952	6.8	2,848	3.2	34,171	3.5	
Asian-East	142,410	6.0	79,375	7.0	749	0.8	15,712	1.6	
Hispanic	1,146,131	48.0	352,828	31.1	55,796	62.7	674,935	69.8	
Maternal age (years)									<0.001
<20	135,601	5.7	23,456	2.1	9,135	10.3	93,719	9.7	
20–24	450,578	18.9	116,979	10.3	25,817	29.0	269,676	27.9	
25–29	635,013	26.6	279,439	24.7	25,726	28.9	274,936	28.4	
30–34	685,773	28.7	414,288	36.6	17,506	19.7	197,600	20.4	
35–29	380,120	15.9	238,789	21.1	8,606	9.7	102,478	10.6	
40+	98,726	4.1	60,189	5.3	2,219	2.5	28,685	3.0	
Maternal educational attainment									<0.001
No formal education	6,925	0.3	315	0.0	1,046	1.2	5,346	0.6	
8th grade or less	112,242	4.7	6,830	0.6	8,344	9.4	93,520	9.7	
9th grade through 12th grade – no diploma	277,624	11.6	30,968	2.7	16,908	19.0	215,209	22.3	
High school graduate or GED completed	584,464	24.5	177,355	15.7	31,315	35.2	332,376	34.4	
Some college credit – no degree	467,771	19.6	219,743	19.4	18,152	20.4	191,023	19.8	
Associate degree	150,082	6.3	92,503	8.2	4,470	5.0	38,215	4.0	
Bachelor’s degree	436,961	18.3	340,088	30.0	4,380	4.9	44,044	4.6	
Master’s degree	184,179	7.7	156,745	13.8	773	0.9	8,042	0.8	
Doctorate or professional degree	62,140	2.6	56,159	5.0	136	0.2	2,005	0.2	
Unknown or not stated	103,423	4.3	52,434	4.6	3,485	3.9	37,314	3.9	
Types of payment sources for prenatal care									
Medi-Cal without CPSP support services	967,094	40.5							
Other government programs	77,994	3.3							
Private insurance company	1,133,140	47.5							
Self-pay	83,190	3.5							
Medi-Cal with CPSP support services	89,009	3.7							
Other	24,961	1.1							
Unknown/unreported pay source	10,423	0.4							
Pre-pregnancy smoking									<0.001
None	2,328,650	97.6	1,121,971	99.0	84,174	94.6	930,912	96.3	
≥1 cigarettes/day	41,395	1.7	8,649	0.8	4,265	4.8	25,110	2.6	
unknown smoking status	15,766	0.7	2,520	0.2	570	0.6	11,072	1.1	
Parity									<0.001
One	937,737	39.3	505,957	44.7	30,185	33.9	320,043	33.1	
2–4	1,332,884	55.9	601,621	53.1	52,435	58.9	572,897	59.2	
5–9	109,522	4.6	24,416	2.2	6,239	7.0	71,966	7.4	
10+	1,993	0.1	349	0.0	93	0.1	1,418	0.2	
unknown	3,675	0.2	797	0.1	57	0.1	770	0.1	
BMI (kg/m^2^)									<0.001
<18.5	90,736	3.8	42,044	3.7	2,983	3.4	33,807	3.5	
18.5–24.9	1,088,545	45.6	583,334	51.5	33,349	37.5	372,966	38.6	
25–29.9	595,503	25.0	264,714	23.4	24,145	27.1	266,019	27.5	
30–34.9	510,583	21.4	204,522	18.1	24,797	27.9	253,853	26.3	
unknown	100,444	4.2	38,526	3.4	3,735	4.2	40,449	4.2	
Pre-pregnancy diabetes									<0.001
No	2,373,190	99.5	1,128,022	99.6	88,565	99.5	960,719	99.3	
Yes	12,621	0.5	5,118	0.5	444	0.5	6,375	0.7	
Gestational diabetes									<0.001
No	2,254,142	94.5	1,069,686	94.4	83,099	93.4	912,549	94.4	
Yes	131,669	5.5	63,454	5.6	5,910	6.6	54,545	5.6	
Pre-pregnancy hypertension (Chronic)									<0.001
No	2,367,991	99.3	1,123,242	99.1	88,282	99.2	960,798	99.4	
Yes	17,820	0.8	9,898	0.9	727	0.8	6,296	0.7	
Gestational hypertension (PIH, Pre-eclampsia)									<0.001
No	2,306,837	96.7	1,093,080	96.5	86,320	97.0	935,175	96.7	
Yes	78,974	3.3	40,060	3.5	2,689	3.0	31,919	3.3	
Month of birth									<0.001
January	194,797	8.2	90,277	8.0	7,423	8.3	81,050	8.4	
February	180,461	7.6	84,970	7.5	6,765	7.6	74,298	7.7	
March	196,742	8.3	94,380	8.3	7,451	8.4	78,895	8.2	
April	187,562	7.9	90,654	8.0	6,994	7.9	74,834	7.7	
May	195,627	8.2	95,154	8.4	7,443	8.4	77,278	8.0	
June	191,982	8.1	92,382	8.2	7,100	8.0	76,656	7.9	
July	206,366	8.7	98,361	8.7	7,671	8.6	83,336	8.6	
August	216,292	9.1	102,632	9.1	7,774	8.7	87,997	9.1	
September	212,966	8.9	100,814	8.9	7,961	8.9	86,403	8.9	
October	208,180	8.7	98,419	8.7	7,726	8.7	84,853	8.8	
November	195,555	8.2	92,146	8.1	7,052	7.9	79,724	8.2	
December	199,281	8.4	92,951	8.2	7,649	8.6	81,770	8.5	
Year of birth									<0.001
2012	485,301	20.3	225,220	19.9	19,168	21.5	205,907	21.3	
2013	475,179	19.9	222,536	19.6	19,242	21.6	195,683	20.2	
2014	483,116	20.3	229,982	20.3	17,308	19.5	192,945	20.0	
2015	472,648	19.8	228,360	20.2	16,261	18.3	188,411	19.5	
2016	469,567	19.7	227,042	20.0	17,030	19.1	184,148	19.0	

California county-level averages of the ^a^unemployment rate in the 12-months prior to birth, ^b^proportion of foreign born population in 5-year period prior to birth year, ^c^proportion of non-English speaking population in the 5-year period prior to birth year. ^d^Pacific Islander group includes Hawaiian, Guamanian, and Samoan groups. ^e^P-values compare the private insurance, Medi-Cal with CPSP, and Medi-Cal without CPSP prenatal care payment source groups.

The crude proportion of birth outcomes significantly differed between payment sources for prenatal care (Table 2). Those with unknown/unreported source of payment for care had the highest proportions of adverse outcomes: PTB: (16.2%), SPTB: (12.6%), LBW (9.2%), Medi-Cal without CPSP (PTB: 7.6%, SPTB: 4.6%, LBW: 5.8%), those with “other” source (PTB: 6.9%, SPTB: 3.9%; LBW: 5.2%), those with “other” government programs (PTB: 6.6%, SPTB: 4.2%, LBW: 4.9%), those with private insurance (PTB: 6.4%; SPTB: 3.9%; LBW: 4.8%) and the Medi-Cal-CPSP (PTB: 6.4%; SPTB: 4.1%; LBW: 4.7%). Those with the “self-pay” category had the lowest ratio of adverse birth outcomes (PTB: 4.7%, SPTB: 3.9%, LBW: 3.6%).

**Table 2:** Frequencies (%) of outcomes by payment source for prenatal care.

	Medi-Cal without CPSP	Other govt. progs.	Private insurance	Self-pay	Medi-Cal with CPSP	Other	Unknown/ unreported source	
	N(%)	N(%)	N(%)	N(%)	N(%)	N(%)	N(%)	*p*
Preterm	73,163	5,135	72,746	3,895	5,662	1,714	1,690	<0.001
	(7.6%)	(6.6%)	(6.4%)	(4.7%)	(6.4%)	(6.9%)	(16.2%)	
Spontaneous preterm	43,526	3,178	43,483	2,498	3,581	931	1,254	<0.001
	(4.6%)	(4.2%)	(3.9%)	(3.1%)	(4.1%)	(3.9%)	(12.6%)	
Low birthweight	55,696	3,843	54,146	3,015	4,217	1,299	946	<0.001
	(5.8%)	(4.9%)	(4.8%)	(3.6%)	(4.7%)	(5.2%)	(9.2%)	

For each outcome, each N (%) figures represents positive cases for the outcome within the insurance subgroup.

Our three multilevel regressions using the full statewide BSMF sample (Table 3) showed that Medi-Cal with CPSP cases had lower likelihood of PTB [adjusted Odds ratio (aOR) = 0.89, 95% confidence interval (CI): 0.86, 0.92], SPTB (aOR = 0.89, 95% CI: 0.86, 0.92), and LBW (aOR = 0.95, 95% CI: 0.92, 0.99) than those with private insurance. The aORs for the association of Medi-Cal with CPSP and the outcomes pushed the point estimates for the outcomes of PTB (OR = 0.98, 95% CI: 0.95, 1.01) and sPTB (OR = 1.03, 95% CI: 1.00, 1.07) back against null by ~10%. The three Medi-Cal models (Table 4) suggested that Medi-Cal with CPSP had lower odds of PTB (aOR = 0.82, 95% CI: 0.80, 0.85), SPTB (aOR = 0.86, 95% CI: 0.82, 0.89) and LBW (aOR = 0.81, 95% CI: 0.78, 0.84) than those Medi-Cal cases without CPSP (Table 4). The results for the sensitivity analyses within the Medi-Cal subgroup using inverse probability weighting for CPSP treatment selection (see Supplementary Table S1) were similar to the unweighted results in Table 4.

**Table 3:** Multilevel logistic regression results for the association between CPSP and birth outcomes in California.

	Preterm birth		Spontaneous preterm birth		Low birthweight	
	OR (95% CI)	*p*	OR (95% CI)	*p*	OR (95% CI)	*p*
Source of prenatal care (private insurance as reference)						
Medi-Cal + CPSP	0.98 (0.95, 1.01)	0.152	1.03 (1.00, 1.07)	0.077	1.01 (0.98, 1.05)	0.454
Medi-Cal–no CPSP	1.17 (1.16, 1.18)	<0.001	1.17 (1.16, 1.19)	<0.001	1.21 (1.19, 1.22)	<0.001
Other public	1.03 (1.00, 1.07)	0.031	1.10 (1.06, 1.14)	<0.001	1.05 (1.02, 1.09)	0.004
Self-pay	0.71 (0.68, 0.73)	<0.001	0.77 (0.74, 0.81)	<0.001	0.75 (0.72, 0.77)	<0.001
Other	1.07 (1.02, 1.12)	0.011	0.98 (0.92, 1.05)	0.501	1.08 (1.02, 1.14)	0.009
	aOR (95% CI)	*p*	aOR (95% CI)	*p*	aOR (95% CI)	*p*
Source of prenatal care (private insurance as reference)						
Medi-Cal + CPSP	0.89 (0.86, 0.92)	<0.001	0.89 (0.86, 0.92)	<0.001	0.95 (0.92, 0.99)	0.007
Medi-Cal–no CPSP	1.05 (1.04, 1.07)	<0.001	1.01 (0.99, 1.03)	0.307	1.14 (1.12, 1.16)	<0.001
Other public	1.04 (1.00, 1.07)	0.029	1.03 (0.99, 1.07)	0.105	1.05 (1.01, 1.09)	0.007
Self-pay	0.81 (0.78, 0.84)	<0.001	0.82 (0.78, 0.85)	<0.001	0.83 (0.80, 0.87)	<0.001
Other	1.02 (0.97, 1.08)	0.367	0.91 (0.85, 0.97)	0.005	1.03 (0.98, 1.10)	0.253
Race/ethnicity (Non-Hispanic Whites as reference)						
Black	1.61 (1.57, 1.64)	<0.001	1.51 (1.47, 1.56)	<0.001	2.33 (2.27, 2.39)	<0.001
Hispanic	1.21 (1.19, 1.23)	<0.001	1.24 (1.21, 1.26)	<0.001	1.30 (1.28, 1.33)	<0.001
Asian-East	0.92 (0.90, 0.95)	<0.001	0.99 (0.95, 1.02)	0.510	1.09 (1.05, 1.12)	<0.001
Asian-Southeast	1.50 (1.47, 1.54)	<0.001	1.56 (1.52, 1.61)	<0.001	1.87 (1.82, 1.92)	<0.001
Asian-South	1.31 (1.26, 1.36)	<0.001	1.21 (1.15, 1.26)	<0.001	2.31 (2.23, 2.39)	<0.001
Asian Other	1.22 (1.14, 1.31)	<0.001	1.30 (1.20, 1.42)	<0.001	1.55 (1.44, 1.67)	<0.001

County of maternal residence was used as the Level 2 cluster. Individual-level maternal covariates included race/ethnicity, age, education, pre-pregnancy/gestational diabetes, pre-pregnancy/gestational hypertension, cigarette smoking during pregnancy, pre-pregnancy BMI, parity, birth month, birth year. County-level covariates included average unemployment rate for the 12 months prior to birth, 5-year average proportion of foreign-born prior to year of birth year, and 5-year average proportion of non-English speakers prior to birth year.

**Table 4:** Multilevel logistic regression results for the association between CPSP and birth outcomes in California (Medi-Cal subgroup analyses).

	Preterm birth		Spontaneous preterm birth		Low birthweight	
	OR (95% CI)	*p*	OR (95% CI)	*p*	OR (95% CI)	*p*
Source of payment for prenatal care (Medi-Cal without CPSP as reference)						
Medi-Cal + CPSP	0.82 (0.79, 0.85)	<0.001	0.86 (0.83, 0.89)	<0.001	0.82 (0.79, 0.85)	<0.001
	aOR (95% CI)	*p*	aOR (95% CI)	*p*	aOR (95% CI)	*p*
Source of payment for prenatal care (Medi-Cal without CPSP as reference)						
Medi-Cal + CPSP	0.82 (0.80, 0.85)	<0.001	0.86 (0.82, 0.89)	<0.001	0.81 (0.78, 0.84)	<0.001
Race/ethnicity (Non-Hispanic Whites as reference)						
Black	1.46 (1.41, 1.51)	<0.001	1.40 (1.34, 1.46)	<0.001	2.10 (2.02, 2.17)	<0.001
Hispanic	1.08 (1.05, 1.11)	<0.001	1.12 (1.09, 1.16)	<0.001	1.11 (1.08, 1.15)	<0.001
Asian-East	0.78 (0.73, 0.84)	<0.001	0.91 (0.83, 0.99)	0.032	0.84 (0.77, 0.91)	<0.001
Asian-Southeast	1.24 (1.18, 1.29)	<0.001	1.33 (1.26, 1.41)	<0.001	1.48 (1.41, 1.55)	<0.001
Asian-South	1.14 (1.05, 1.25)	0.002	1.02 (0.91, 1.15)	0.695	1.61 (1.47, 1.75)	<0.001
Asian other	1.06 (0.94, 1.21)	0.353	1.13 (0.97, 1.32)	0.127	1.29 (1.12, 1.48)	<0.001

County of maternal residence was used as the Level 2 cluster. Individual-level maternal covariates included race/ethnicity, age, education, pre-pregnancy/gestational diabetes, pre-pregnancy/gestational hypertension, cigarette smoking during pregnancy, pre-pregnancy BMI, parity, birth month, birth year. County-level covariates included average unemployment rate for the 12 months prior to birth, 5-year average proportion of foreign-born prior to year of birth year, and 5-year average proportion of non-English speakers prior to birth year.

In both the full-sample analysis and the Medi-Cal subset analyses, persons from minority racial/ethnic groups had significantly higher risk than non-Hispanic White persons, with the exception of persons from the East Asian group who had significantly lower risk than non-Hispanic White persons in four of the six models (For the PTB outcome in full sample: aOR = 0.92, 95% CI: 0.90, 0.95; and in the Medi-Cal subgroup for all outcomes PTB: aOR = 0.78, 95% CI: 0.73, 0.84; SPTB: aOR = 0.91, 95% CI: 0.83, 0.99; LBW: aOR = 0.84, 95% CI: 0.77, 0.91).

In all our multilevel models, the random effect for county was significant (*p* < 0.001) indicating that the two-level models differed significantly from their single-level counterparts. The interaction terms between race/ethnicity and CPSP enrollment in the Medi-Cal models were all insignificant indicating that the protective effect of CPSP did not vary across these minority racial/ethnic groups. Therefore, only the main effect of CPSP has been presented in Table 4.

## Discussion

Using California’s statewide data, we identified a consistent protective effect of Medi-Cal’s CPSP on PTB, SPTB and LBW, after adjusting for both the county-level and individual-level covariates. we strengthened the evidence from the two earlier studies whereby pilot projects of CPSP demonstrated protective effects (1, 18). Our study provided important information for stakeholders of maternal and child health, as preterm birth and low birthweight are known to be very substantial burdens for families and societies worldwide (38).

Despite the substantial differences in PTB and LBW risk across racial/ethnic groups, we found the association between CPSP and birth outcome did not vary across racial/ethnic subgroups, a finding consistent with previous evidence that increasing access to prenatal service led to better outcomes but not smaller racial disparity (39). It remained unclear how well CPSP was understood by and how accessible it was for Medi-Cal beneficiaries of minority background. In our sample, only 8.4% of the Medi-Cal cases were covered by CPSP (see Supplementary Figure S1 for CPSP enrollment across California State counties), signaling potential room for improvement in promotion and dissemination. Given the excess risk for PTB and LBW among persons from Southeast Asian and South Asian race/ethnicity groups as shown in our analyses, it might be particular importance to develop culturally and linguistically appropriate program promotion to these Asian subgroups, as a lack of cultural affinity plus the myth of Asian American persons as “the model minority” group (40) could compromise the CPSP uptake rate among Southeast Asian and South Asian persons.

It might be plausible to think that all key components of CPSP (health education, nutritional supplements and psychosocial counseling plus referrals if necessary) might have contributed to the observed risk reduction of adverse birth outcomes through their separate intervention mechanisms as documented by perinatology literature, while it could also be plausible that these different components might have produced a synergistic benefit that a standalone intervention could not produce by itself [for example, there could be a synergy between health education classes about optimal nutritional intake during pregnancy and the actual delivery of nutritional supplements to those who have received nutritional health education (41)]. So far, the evidence of health education’s protective effect against adverse birth outcomes seems to be the strongest in the field of antenatal nutritional education: a 2015 review found that nutritional education programs with the aim of increasing energy and protein intake among pregnant women appears to be effective in reducing the risk of PTB (two trials, 449 women) and LBW (one trial, 300 women) (41). The effectiveness of psychosocial support on reducing LBW, meanwhile, has been supported by a 2015 meta-analysis about four trials among psychosocial interventions among teenage pregnant women (42). The evidence for the effectiveness of psychosocial interventions alone on the PTB outcome and birth outcomes among women of other age groups remain inconclusive. As a possible piece of circumstantial evidence for the effectiveness of health education and psychosocial support in lowering PTB and LBW, it has been noted that the low level of PTB and LBW observed in “birth centers” [a holistic midwifery model of care (43)] might be attributable to its substantial time to education and psychosocial support, to an extent typically unavailable in usual prenatal visits (43).

Compared with CPSP’s service in health education and psychosocial support, its delivery of nutritional supplements to the pregnant women has more direct support from epidemiological literature with high level of evidence strength. For example, a 2018 meta-analysis of randomized controlled trials (4,193 women) summarized that prenatal supplementation of Omega-3 fatty acid led to a 58% reduction of early preterm birth risk and 17% reduction of any premature birth risk compared to those in the placebo groups (44), and its subgroup analysis found that these protective effects did not vary by women’s risk status, dosage of supplement or timing of the intervention. As for the outcome of LBW, a systematic review of 30 trials concluded that supplementing pregnant women with vitamin D alone probably reduces the risk of LBW but not PTB (45), while a meta-analysis shows that balanced energy and protein supplementation reduce the risk of infants born small-for-gestational age (Relative risk: 0.79, 95% CI: 0.69–0.90, 4,408 women, moderate-quality evidence) (41). Furthermore, a meta-analysis of prenatal iron use has been found to reduce the risk of LBW (Relative risk: 0.81, 95% CI: 0.71–0.93, 13 trials) but not PTB. These results, however, need to be interpreted with caution since a modification of the proven nutritional supplementation intervention might actually change the intervention’s protective effects into net harm: the same review that found the benefit of balanced energy and protein supplementation also found that high-protein supplementation may be harmful to the fetus (41), while the review that found the benefits of Vitamin D supplementation also found that supplementation with vitamin D plus calcium may increase the PTB risk (Relative risk: 1.52, 95% CI: 1.01–2.28; 5 trials, 942 women, low-certainty evidence) (45). For the specific context of our study about California’s CPSP service, though, the fact that CPSP’s prenatal assessment/reassessment and individualized care plan included screening questions about the woman’s iron and vitamin intake is a clue why CPSP enrollment might be associated with lower risk for PTB and LBW.

Our study is limited in that no causal inference can be drawn from the observed link between CPSP and outcome due to the voluntary nature of CPSP participation. In other words, women who decided to enroll in CPSP could have certain protective factors that reduced risk for preterm births and low birthweight, factors that might not be available from our observational data. Moreover, the BSMF dataset did not have the frequency of CPSP service utilization and therefore we could not test the effect of the CPSP utilization frequency on the outcomes. Also, details about the type of CPSP service (education, nutritional supplements, and psychosocial counseling plus referrals if necessary) utilized were not available, and therefore we were unable to tell which component of the CPSP played a significant role in PTB and LBW risk reduction. Also, there is a lack of data on the extent to which the program was implemented in healthcare settings. It is possible that some women may not have enrolled due to lesser access to the program (17). Therefore, more in-depth detailed study (such as survey questionnaires with more specific questions about care utilization behavior among the CPSP enrollees as well as non-CPSP enrollees) is needed to understand which CPSP component might have contributed to outcome improvement.

Finally, while it might be a promising sign to see the significantly negative association between CPSP enrollment and spontaneous PTB, the type of preterm births that has been known as hard to understand and thus difficult to prevent, it is also important to note that the classification of preterm subtype from birth registries such as California’s BSMF might not be perfectly accurate as the methodology of accurately categorizing types of preterm births is still under development (46) and has yet to reach perfection.

Nevertheless, it is encouraging to see that the CPSP cases had better birth outcomes than those covered by private insurance after adjusting for confounding factors. As the evidence for Medicaid expansion’s benefits on birth outcome remained inconclusive as of 2022 (47), population health stakeholders might consider strengthening the reach of comprehensive perinatal services like CPSP so that insurance coverage could have a stronger benefit on vitally important health outcomes such as preterm birth and low birthweight.

## Data availability statement

The county level data used in this study is from publicly accessible sources (Bureau of Labor Statistics: https://www.bls.gov/ and County Health Ranking: https://www.countyhealthrankings.org/). Other data analyzed in this study is subject to the following licenses/restrictions: The California Birth Data used in our study is restricted and the authors in this study do not have permission to share it. Requests to access these datasets should be directed to https://www.cdph.ca.gov.

## Ethics statement

The studies involving humans were approved by Clemson University IRB; the Committee for the Protection of Human Subjects (CPHS); and the Vital Statistics Advisory Committee (VSAC). The studies were conducted in accordance with the local legislation and institutional requirements. Written informed consent for participation was not required from the participants or the participants’ legal guardians/next of kin in accordance with the national legislation and institutional requirements.

## Author contributions

SL: Conceptualization, Data curation, Formal analysis, Writing – original draft, Writing – review & editing. AS: Writing – review & editing. LC: Writing – review & editing. JL: Writing – review & editing. LM: Conceptualization, Funding acquisition, Writing – review & editing.
