# Supplementary material for: California’s Comprehensive Perinatal Services Program and birth outcomes
Source: Front Public Health. 2023 Dec 21;11:1321313. doi: 10.3389/fpubh.2023.1321313 (PMC10764413; doi:10.3389/fpubh.2023.1321313)
Supplement: Supplementary file 1 [file Table_1.DOCX]

Supplementary Material

## Supplementary Figure


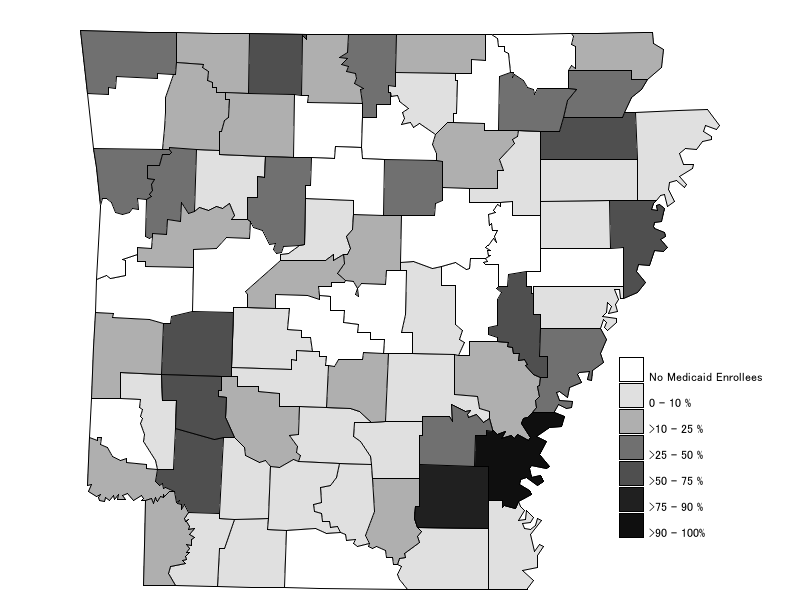


**Figure S1.** California State Map depicting the level of CPSP (Comprehensive Perinatal Services Program) enrollment among pregnant Medicaid women with singleton live births within each county from 2012-2016. This map has been produced using data from California Birth Statistical Master Files. The map legend shows the color scheme used to indicate different classes of the within-county percentage of CPSP enrollment among pregnant Medicaid women.

## Supplementary Table

| **Table S1: Multilevel Logistic Regression Results for the Association Between CPSP and Birth Outcomes in California using Inverse Probability Weighting for CPSP selection** | | | | | | |
| --- | --- | --- | --- | --- | --- | --- |
|  | **Preterm Birth** | | **Spontaneous Preterm Birth** | | **Low Birthweight** | |
|  | **OR (95% CI)** | **p** | **OR (95% CI)** | **p** | **OR (95% CI)** | **p** |
| ***Source of payment for prenatal care (Medi-Cal without CPSP as reference)*** |  |  |  |  |  |  |
| Medi-Cal+CPSP | 0.89 (0.86, 0.91) | <.001 | 0.92 (0.89, 0.95) | <.001 | 0.87 (0.84, 0.90) | <.001 |
|  | **aOR (95% CI)** | **p** | **aOR (95% CI)** | **p** | **aOR (95% CI)** | **p** |
| ***Source of payment for prenatal care (Medi-Cal without CPSP as reference)*** |  |  |  |  |  |  |
| Medi-Cal+CPSP | 0.86 (0.83, 0.89) | <.001 | 0.88 (0.85, 0.92) | <.001 | 0.84 (0.81, 0.87) | <.001 |
| ***Race/ethnicity (Non-Hispanic Whites as reference)*** |  |  |  |  |  |  |
| Black | 1.47 (1.42, 1.52) | <.001 | 1.42 (1.36, 1.48) | <.001 | 2.11 (2.04, 2.19) | <.001 |
| Hispanic | 1.09 (1.06, 1.12) | <.001 | 1.13 (1.09, 1.16) | <.001 | 1.12 (1.09, 1.15) | <.001 |
| Asian-East | 0.79 (0.74, 0.85) | <.001 | 0.94 (0.86, 1.03) | .171 | 0.85 (0.79, 0.92) | <.001 |
| Asian-Southeast | 1.24 (1.19, 1.30) | <.001 | 1.35 (1.28, 1.43) | <.001 | 1.50 (1.43, 1.58) | <.001 |
| Asian-South | 1.15 (1.06, 1.25) | .001 | 1.05 (0.94, 1.18) | .403 | 1.63 (1.50, 1.78) | <.001 |
| Asian Other | 1.07 (0.94, 1.21) | .311 | 1.15 (0.98, 1.34) | .080 | 1.30 (1.13, 1.48) | <.001 |
| Note: CPSP (Comprehensive Perinatal Services Program). County of maternal residence was used as the Level 2 cluster. Individual-level maternal covariates included race/ethnicity, age, education, pre-pregnancy/gestational diabetes, pre-pregnancy/gestational hypertension, cigarette smoking during pregnancy, pre-pregnancy BMI, parity, birth month, birth year. County-level covariates included average unemployment rate for the 12 months prior to birth, 5-year average proportion of foreign-born prior to year of birth year, and 5-year average proportion of non-English speakers prior to birth year. | | | | | | |
